# Bladder Mucosal CO_2_ Compared with Gastric Mucosal CO_2_ as a Marker for Low Perfusion States in Septic Shock

**DOI:** 10.1100/2012/360378

**Published:** 2012-04-19

**Authors:** Gemma Seller-Pérez, Manuel E. Herrera-Gutiérrez, Cesar Aragón-González, Maria M. Granados, Juan M. Dominguez, Rocío Navarrete, Guillermo Quesada-García, Juán Morgaz, Rafael Gómez-Villamandos

**Affiliations:** ^1^Intensive Care Medicine, University Hospital Carlos Haya, 29010 Málaga, Spain; ^2^Veterinary Antesthesiology, Veterinary Teaching Hospital, University of Córdoba, 14071 Córdoba, Spain

## Abstract

Recent reports indicate the possible role of bladder CO_2_ as a marker of low perfusion states. To test this hypothesis, shock was induced in six beagle dogs with 1 mg/kg of *E. coli* lipopolysaccharide, gastric CO_2_ (CO_2_-G) was measured with a continuous monitor, and a pulmonary catheter was inserted in the bladder to measure CO_2_ (CO_2_-B). Levels of CO_2_-B were found to be lower than those of CO_2_-G, with a mean difference of 36.8 mmHg (*P* < 0.001), and correlation between both measurements was poor (*r* ^2^ = 0.16). Even when the correlation between CO_2_-G and ΔCO_2_-G was narrow (*r* ^2^ = 0.86), this was not the case for the relationship between CO_2_-B and ΔCO_2_-B (*r* ^2^ = 0.29). Finally, the correlation between CO_2_-G and base deficit was good (*r* ^2^ = 0.45), which was not the case with the CO_2_-B correlation (*r* ^2^ = 0.03). In our experience, bladder CO_2_ does not correlate to hemodynamic parameters and does not substitute gastric CO_2_ for detection of low perfusion states.

## 1. Introduction

Tissue pressure of CO_2_ is considered to be an indicative parameter of the microvascular perfusion, and this measure can be obtained from highly vascular areas such as sublingual, brain, conjunctiva, skin, or typically the intestinal area [1].

The most widespread indication is the estimation of tissue perfusion in sepsis and septic shock, although its use has been described in other types of shock (especially the hemorrhagic) [2], cardiovascular surgery, transplantation, or trauma and is associated with outcome [3].

Most commonly explored areas are the sublingual and intestinal areas [4, 5] but theoretically any sufficiently vascularized area would be suitable for measurement. We intend to study the value of bladder intramucosal CO_2_ in an animal model of induced sepsis.

## 2. Material and Methods

### 2.1. Anesthetic Protocol

The anesthesiologists of this centre controlled the subjects. We studied six Beagle dogs between 12 and 15 Kg of weight that underwent the usual anesthetic procedure in this centre: anesthesia with sevoflorane, relaxation with atracurium, and mechanical ventilation aimed to obtain normal PaCO_2_.

### 2.2. Monitoring

All animals were monitored with a Picco catheter in the femoral artery, a central venous jugular vein catheter, and continuous end-tidal CO_2_ measurement. In all subjects we registered every 60 minutes the mean arterial pressure (MAP), cardiac output (CO), and systolic volume variability (SVV). Arterial blood gases were measured before sepsis and then every 60 minutes for six hours. Survivors at this time were euthanized following the standard measured approved for this veterinary hospital.

Measurement of gastric mucosa CO_2_ (CO_2_-G) used for the calculation of the gap between PaCO_2_ and CO_2_-G (ΔCO_2_-G) was performed continuously with a Datex Ohmeda (GE Healthcare) monitor tonometry module and the probe for gastric tonometry TRIP Catheter (Intrumentarium Corp) that was positioned by gastroscopy after induction of anesthesia.

Measurement of CO_2_ in bladder mucosa (CO_2_-B) used for the calculation of the gap between PaCO_2_ and CO_2_-B (ΔCO2-B) was performed in agreement with the technique described by Fiddian-Green [6] through a pulmonary artery catheter inserted into the bladder infusing 1 mL saline in the balloon and maintaining this for 60 minutes prior to its removal with subsequent immediate analysis to determine the pressure of CO_2_.

### 2.3. Provocation of Sepsis [7]

With the subjects anesthetized, monitored in stable condition, we infused 1 mgr/Kg of body weight of ultra pure Escherichia Coli Lipopolisacharid (strain 0111 : B4, InvivoGen) diluted in 20 mL of saline solution and infused in 10 minutes.

### 2.4. Ethical Issues

This study was carried out in an experimental operating room of the Veterinary Hospital of the University of Córdoba, Spain. Survivors at the end of the follow-up were euthanized following the standard measures approved for this veterinary hospital. The Ethics and Clinical Research Committee at the Veterinary Hospital of the University of Córdoba have approved this protocol. This research was funded by a nonrestricted grant by Hospal with complete independence of the results of the study. None of the authors have any commercial relationship with the funding company.

### 2.5. Statistical Analysis

The analysis was carried out by the statistical package SPSS for Windows 11. Data are shown as mean ± standard deviation or proportions. For comparisons we used the Pearson correlation test and scatter plots, with a level of significance for all tests of 0.05.

## 3. Results

**Figure 1:**
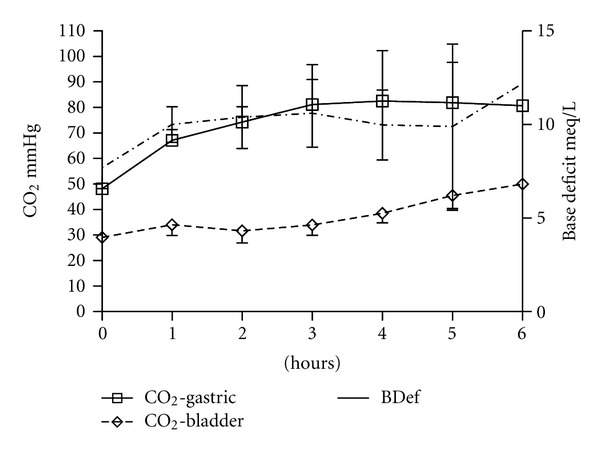
Changes of gastric and bladder mucosal CO_2_ and base deficit for six hours after septic shock provocation.

In all the subjects a state of severe shock was evident at the end of the induction. The subsequent evolution was marked by the maintenance of the low perfusion status until the end of the experiment (Table 1) and this effect was detected by changes in base deficit and CO_2_-G but not so in CO_2_-B (Figure 1).

**Figure 2:**
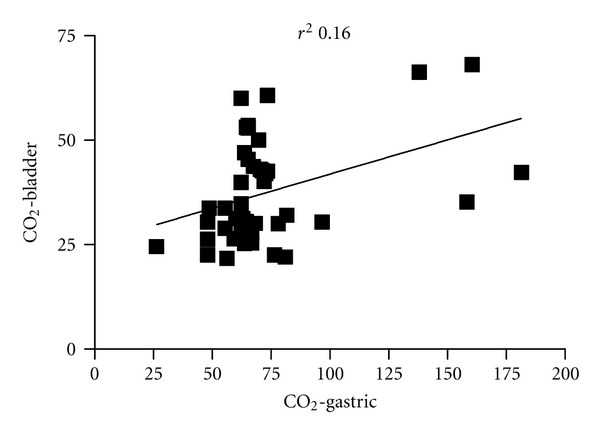
Relationship between gastric mucosal CO_2_ and bladder mucosal CO_2_.

**Figure 3:**
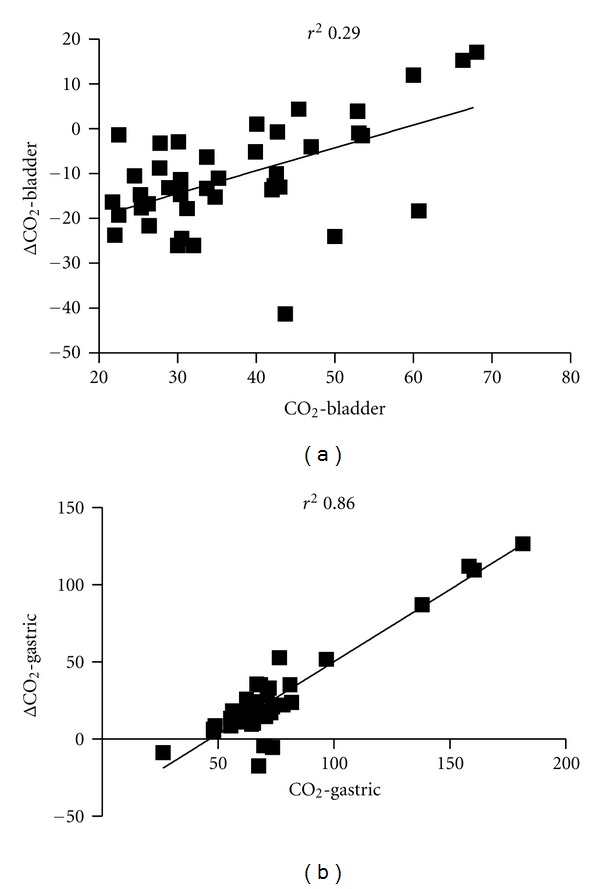
Correlation between mucosal CO_2_ and Gap between mucosal and arterial CO_2_.

**Figure 4:**
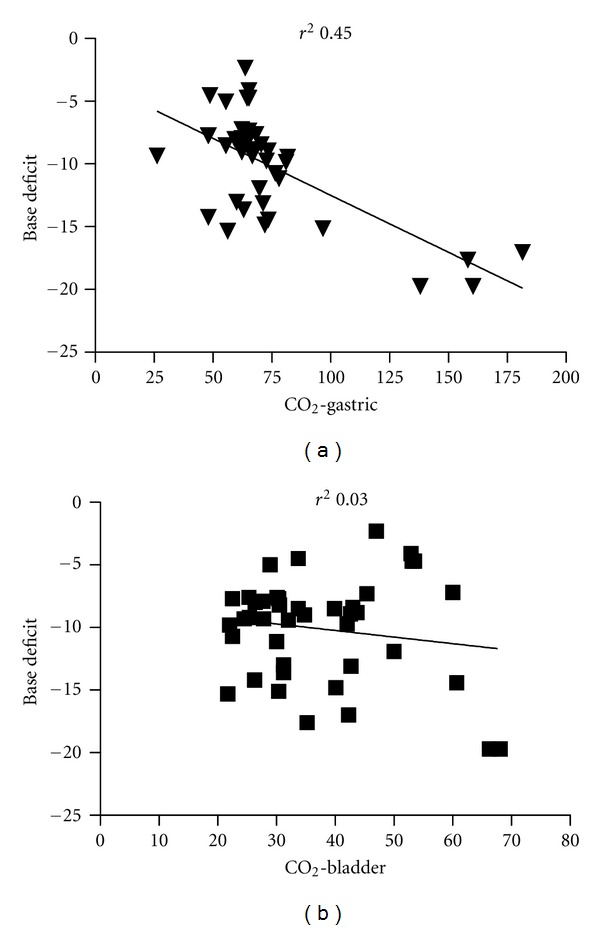
Correlation between mucosal (gastric and bladder) CO_2_ and base deficit in arterial blood.

In all measures, levels of CO_2_-B were found to be significantly lower than CO_2_-G, with a mean difference of 36.8 (*P* = 0.001)  mmHg. Even though a correlation was found between both measurements (Figure 2), this was poor (*r* ^2^ = 0.29). In the same way, even when the correlation between CO_2_-G and ΔCO_2_-G was narrow (as it was expected), this was not the case for the relationship between CO_2_-B and ΔCO_2_-B (Figure 3). Finally, the correlation between CO_2_-G and base deficit was good (*r* ^2^ = 0.45); this however was not the case for the CO_2_-B correlation (*r* ^2^ = 0.03) (Figure 4).

## 4. Discussion

Determination of the mucosal CO_2_ is one accepted method for assessment of perfusion states in shock patients. The possibility to measure this parameter in the bladder presents as an attractive alternative because this is a virtually universal access in patients in shock. However, according to our results, the usefulness of the bladder mucosal CO_2_ as estimator of low perfusion is limited and cannot replace the accepted determination in gastric mucosa.

Sepsis is one of the major pathologies of intensive care (ICU) and one associated with high morbidity-mortality [8]. An early establishment of an appropriate antimicrobial treatment is the basis of current approaches to its management, but an appropriate resuscitation aimed to improve organs perfusion can have as positive an impact on survival as possible, according to the guidelines of Surviving Sepsis Initiative [9].

To provide an adequate treatment to the septic patient, it is also necessary to count with a proper monitoring protocol, and in this study we have tried to equate our measures to the regular monitoring of the critically ill patient.

The exploration of the perfusion status in the splanchnic area can be a key step in the evaluation of the septic patient if we consider that this is a compromised area (due to the initial compensatory mechanisms against shock) and also a potential source for new inflammatory insults when oxygen delivery is critical. Mucosal cells in the intestinal tract are the most accessible source of information in the abdomen, and evaluating their acid-base status can be a reflection of the adequacy of splanchnic organs perfusion. In terms of the monitoring strategies for the septic patient, the mucosal CO_2_ is an attractive possibility, for both its simplicity and ease of interpretation and is for many clinicians a fundamental monitoring device in critical patients in general and in the septic patient in particular [10, 11], although this debate remains inconclusive because its relationship with the outcome is not unanimously recognized [12]. The possible role of the bladder mucosa as another source for measurement has theoretical benefits, because almost all critically ill patients have a bladder catheter already inserted.

We observed, as expected, a decrease of tissue perfusion starting the first hour after the septic provocation, as measured by CO_2_ in gastric mucosa, whose value was increased gradually over hours, reflecting the increase in hepato-esplachnic blood flow that occurs in sepsis, in concordance with that observed in other studies [13, 14]. This was accompanied by a compatible hemodynamic pattern coincident with that found in other studies of *E. Coli*-induced sepsis in animal models [15].

All the monitoring data obtained in our study are close to those expected in an untreated septic shock. Therefore, our hypothesis was that these changes in low tissue perfusion would also be reflected by data obtained from the bladder tonometry, but this was not the case.

Measurements of CO_2_ obtained from the urinary bladder are not common in clinical practice, although it has been tested on models of ischemia-reperfusion [16] and hemorrhagic shock [17], exhibiting however in the latter case a worse correlation when compared with data provided by the CO_2_ obtained from the gut. These results have been also supported by a study on dogs where the gut PCO_2_ was found to be a more reliable measure when compared with samples measured *in vitro* [18].

While the pathogenesis of sepsis and septic shock is well recognized and it is accepted that the data provided by mucosal CO_2_ have unquestionable validity as a reflection of the severity of the shock, the search for new noninvasive and easy to perform monitoring strategies is constant in this field [19]. This is currently raising more expectation of the probing on the most proximal segments of gastrointestinal tract, especially the sublingual capnography [20].

Our data, in contrast to what might be expected, show that bladder PCO_2_ does not conform to the low perfusion state detected through the multiple monitoring we applied to the subjects of the study. One unavoidable bias in our study regards the use of different methods for gastric and bladder measurements, and this fact could explain in part the differences detected. Notwithstanding, the saline tonometry was the first described [6] and has been used as gold standard for the validation of the air tonometry in gastric mucosa [21]. Until now there have been no studies published with the air tonometry probe into the bladder environment, and its use would have introduced an even greater uncertainty in the results requiring in the first place a validation of the technique in this setting.

Another problem with our study is the small sample of subjects studied. Nevertheless, even with so low number of subjects the difference between the two measures was strikingly evident. Also, this being an experimental study developed in a rigidly controlled scenario, we must presume that under clinical conditions and subjected to more confounding variables, the lack of utility should be even more evident. In any case, a new study in a human clinical environment would definitely clarify this point.

The search for other reproducible and reliable means of measuring tissue CO_2_ should be maintained but, according to our data, measurement through bladder tonometry does not offer meaningful results and should not be used in the clinic as a guide to treatment.

## Figures and Tables

**Table 1:** Evolution of the hemodynamic parameters from basal to end of follow-up.

Minutes	Basal	60	120	180	240	300	360
MAP* mmHg	76 ± 12.6	41.2 ± 5.8	41.8 ± 7.6	39.4 ± 6.4	44.4 ± 12.1	47.1 ± 9.4	44.7 ± 9.4
CO L/min	1.4 ± 0.4	1.07 ± 0.5	1.02 ± 0.5	1.0 ± 0.5	1.06 ± 0.5	1.02 ± 0.5	0.99 ± 0.5
SVV* %	9.7 ± 2.6	18.2 ± 5.3	22.5 ± 5.2	25.5 ± 2.3	23 ± 6.6	21.8 ± 2.9	20.3 ± 4.9
CO_2_-Gastric* mmHg	48.1 ± 12.1	67.1 ± 10.3	74.3 ± 14.9	81.1 ± 38.4	82.5 ± 48.6	81.9 ± 38.7	80.8 ± 28.3
CO_2_-Bladder mmHg	29.1 ± 4.9	34.0 ± 10.4	31.7 ± 11.6	33.9 ± 9.8	38.6 ± 9.4	45.5 ± 14	50.0 ± 14.3
Base Deficit	−7.7 ± 2.8	−10 ± 2.3	−10.5 ± 4.1	−10.6 ± 4.4	−9.9 ± 4.5	−9.8 ± 5.5	−12.0 ± 5.1

*: Statistically significant for *P* < 0.05. MAP: mean arterial pressure; CO: cardiac output; SVV: systolic volume variability. Data as mean ± standard deviation.

## References

[1] Klijn E, Uil D, Bakker J, Ince C (2008). The heterogeneity of the microcirculation in critical illness. *Clinics in Chest Medicine*.

[2] Clavijo-Alvarez J, Sims C, Menconi M (2004). Bladder mucosa pH and PCO_2_ as a minimally invasive monitor of hemorrhagic shock and resuscitation. *Journal of Trauma*.

[3] Levy B, Gawalkiewicz P, Vallet B, Briancon S, Nace L, Bollaert PE (2003). Gastric capnometry with air-automated tonometry predicts outcome in critically ill patients. *Critical Care Medicine*.

[4] Creteur J (2006). Gastric and sublingual capnometry. *Current Opinion in Critical Care*.

[5] Boswell S, Scalea T (2003). Sublingual capnometry: an alternative to gastric tonometry for the management of shock resuscitation. *AACN*.

[6] Fiddian-Green RG (1990). Gut mucosal ischemia during cardiac surgery. *Journal of Thoracic and Cardiovascular Surgery*.

[7] Herrera-Gutiérrez ME, Seller-Pérez G, García GQ, Granados MM, Domínguez JM, Gómez-Villamandos RJ (2011). Development of a septic shock experimental model oriented at training. Application in the training of depuration techniques in the management of severe sepsis. *Medicina Intensiva*.

[8] Annane D (2009). Improving clinical trials in the critically ill: unique challenge-Sepsis. *Critical Care Medicine*.

[9] Dellinger P, Levy M, Carlet J (2008). Surviving Sepsis Campaign: international guidelines for management of severe sepsis and septic shock: 2008. *Intensive Care Medicine*.

[10] Palizas F, Dubin A, Regueira T (2009). Gastric tonometry versus cardiac index as resuscitation goals in septic shock: a multicenter, randomized, controlled trial. *Critical Care*.

[11] Heard S (2003). Gastric tonometry: the hemodynamic monitor of choice (Pro). *Chest*.

[12] Gomersall Ch, Joynt G, Freebairn R, Hung V, Buckley TA, Oh TE (2000). Resuscitation of critically ill patients based on the results of gastric tonometry: a prospective, randomized, controlled trial. *Critical Care Medicine*.

[13] Ackland G, Grocott M, Mythen M (2000). Understanding gastrointestinal perfusion in critical care: so near, and yet so far. *Critical Care*.

[14] Uusaro A, Lahtinen P, Parviainen I, Takala J (2000). Gastric mucosal end-tidal PCO_2_ difference as a continuous indicator of splanchnic perfusion. *British Journal of Anaesthesia*.

[15] Lagoa C, Poli de Fioguereido  L, Cruz RJ, Silva E, Rocha e Silva M (2004). E on splanchnic perfusion in canine model of severe sepsis induced by live Escherichia coli infusion. *Critical Care*.

[16] Lang JD, Evans DJ, DeFigueiredo LP, Hays S, Mathru M, Kramer GC (1999). A novel approach to monitor tissue perfusion: bladder mucosal PCO_2_, PO_2_, and pHi during ischemia and reperfusion. *Journal of Critical Care*.

[17] Dubin A, Pozo O, Edul V (2005). Urinary bladder partial carbon dioxide tension during hemorrhagic shock and reperfusion: an observational study. *Critical Care*.

[18] Boda D, Kaszaki J, Tálosi G (2006). A new simple tool for tonometric determination of the PCO_2_ in the gastrointestinal tract: in vitro and in vivo validation studies. *European Journal of Anaesthesiology*.

[19] Groeneveld A (2000). Tonometry of partial carbon dioxide tension in gastric mucosa: use of saline, buffer solutions, gastric juice or air. *Critical Care*.

[20] Marik P (2005). Regional carbon dioxide monitoring to assess the adequacy of tissue perfusion. *Current Opinion in Critical Care*.

[21] Creteur J, de Backer D, Vincent JL (1997). Monitoring gastric mucosal carbon dioxide pressure using gas tonometry: in vitro and in vivo validation studies. *Anesthesiology*.

